# Molecular Hydrogen Mediates Neurorestorative Effects After Stroke in Diabetic Rats: the TLR4/NF-κB Inflammatory Pathway

**DOI:** 10.1007/s11481-022-10051-w

**Published:** 2022-07-27

**Authors:** Wan-Chao Yang, Ting-ting Li, Qiang Wan, Xin Zhang, Li-Ying Sun, Yu-Rong Zhang, Pei-Chen Lai, Wen-zhi Li

**Affiliations:** 1 Department of Anesthesiology, the Second Affiliated Hospital of Harbin Medical University, Harbin, People’s Republic of China; 2 Institute of Biochemistry and Molecular Biology, College of Medicine, National Taiwan University, Taipei, People’s Republic of China; 3 Asclepius Meditec Co., Ltd, Shanghai, China

**Keywords:** Stroke, Diabetes, Neuroinflammation, Circadian rhythm, Molecular hydrogen

## Abstract

Diabetes is an independent risk factor for stroke and amplifies inflammation. Diabetic stroke is associated with a higher risk of death and worse neural function. The identification of effective anti-inflammatory molecules with translational advantages is particularly important to promote perioperative neurorestorative effects. Applying molecular hydrogen, we measured blood glucose levels before and after middle cerebral artery occlusion (MCAO), 48-h cerebral oedema and infarct volumes, as well as 28-day weight, survival and neurological function. We also measured the levels of TLR4, NF-κB p65, phosphorylated NF-κB p65, catecholamines, acetylcholine and inflammatory factors. All measurements comprehensively showed the positive effect and translational advantage of molecular hydrogen on diabetic stroke. Molecular hydrogen improved the weight, survival and long-term neurological function of rats with diabetic stroke and alleviated changes in blood glucose levels before and after middle cerebral artery occlusion (MCAO), but no difference in circadian rhythm was observed. Molecular hydrogen inhibited the phosphorylation of NF-κB and significantly reduced inflammation. Molecular hydrogen mediates neurorestorative effects after stroke in diabetic rats. The effect is independent of circadian rhythms, indicating translational advantages. The molecular mechanism is related to the TLR4/NF-κB pathway and inflammation.

**Graphical abstract:**

Molecular hydrogen (H_2_) affects outcomes of ischemic stroke with diabetes mellitus (DM).

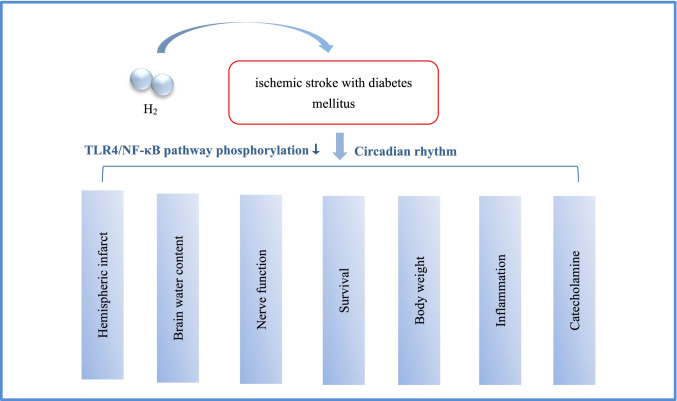

**Supplementary Information:**

The online version contains supplementary material available at 10.1007/s11481-022-10051-w.

## Introduction

Diabetes is an independent risk factor for ischaemic stroke and key factor contributing to increases in its risk and mortality, increasing the challenges of neuro-intensive care (Burchfiel et al. 1994; Goldstein et al. 2001; Janghorbani et al. 2007; Jia et al. 2011; Khoury et al. 2013). Our previous studies have confirmed that molecular hydrogen can effectively improve neurological function and death in stroke mice and effectively reduce cerebral ischemic volume, which has also been subsequently confirmed by multiple sources (Chen et al. 2019; Li et al. 2019; Jiang et al. 2020). However, the manifestation of diabetic exacerbation of stroke and the role and mechanism of molecular hydrogen in diabetic stroke still need to be explored.

The circadian rhythm is an important cause of the failure of many basic research results obtained in stroke models to be translated into clinical treatments. Molecular hydrogen has been a leading anti-inflammatory molecule investigated in recent years. As shown in our previous studies, molecular hydrogen effectively attenuates brain oedema and promotes improved neurological outcomes after TBI (Li et al. 2020). Circadian rhythm is an important cause of the failure of many clinical studies on stroke (Esposito et al. 2020). The present study further comprehensively explored the neuroprotective and anti-inflammatory effects of molecular hydrogen. We innovatively compared circadian rhythms to determine the translational advantage at the same time. We further explored the molecular mechanism of the inflammatory pathway in depth. We aimed to explore the neurorestorative effects of molecular hydrogen after stroke in diabetic rats via the inflammatory pathway and circadian rhythm to obtain a translational advantage.

## Materials and Methods

### Animals

**Fig. 1:**
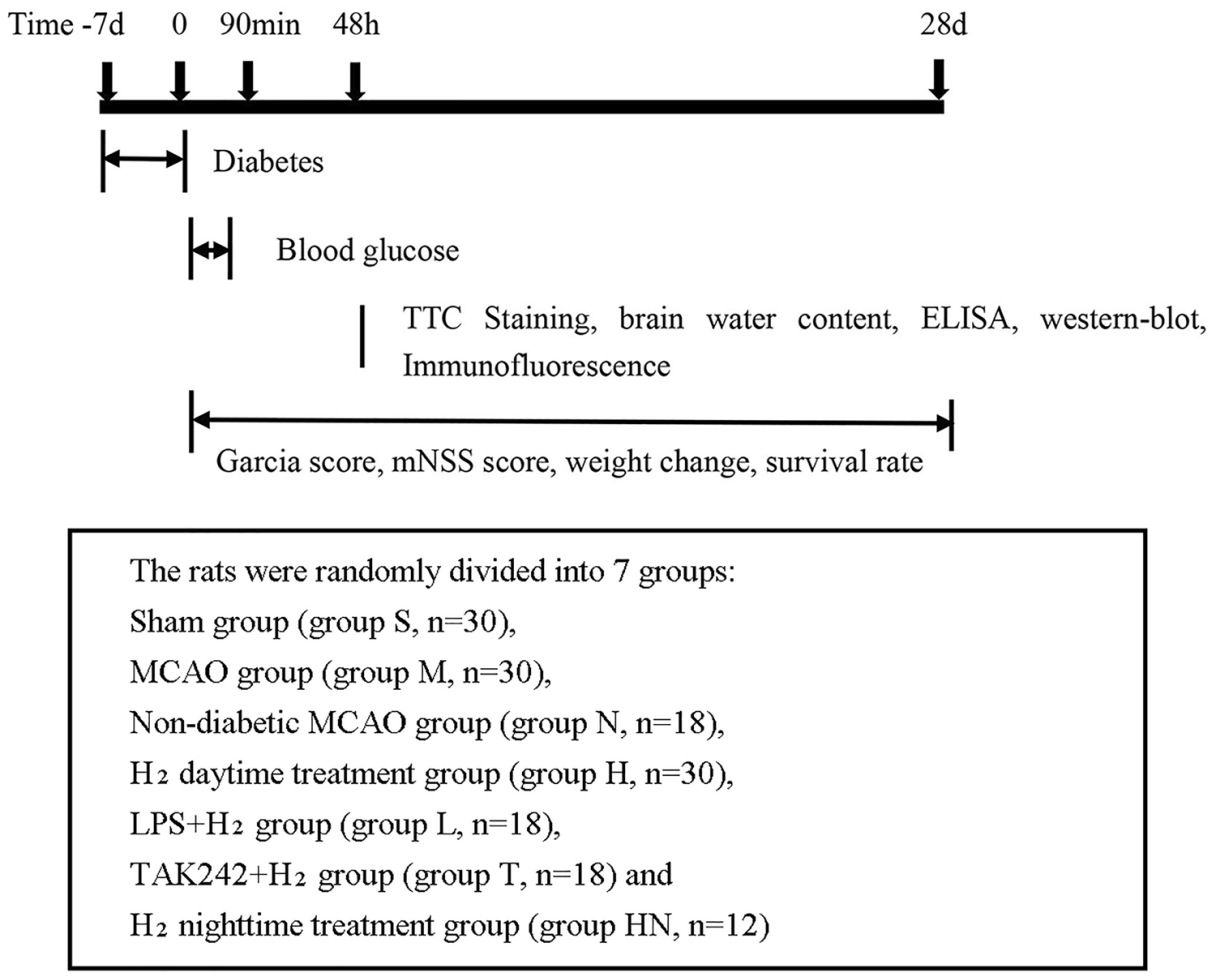
Flow chart. Timeline of the experimental protocol

One hundred fifty-six male Sprague–Dawley (SD) rats weighing 60–80 g were obtained from the Laboratory Animal Department of Harbin Medical University. The rats were reared on a 12 h light–dark cycle under controlled temperature conditions (21–24 °C), and they were provided free access to food and water. The rats were randomly divided into 7 groups: Sham group (group S, n = 30), MCAO group (group M, n = 30), Non-diabetic MCAO group (group N, n = 18), H_2_ daytime treatment group (group H, n = 30), LPS + H_2_ group (group L, n = 18), TAK242 + H_2_ group (group T, n = 18) and H_2_ night-time treatment group (group HN, n = 12) (Fig. 1). According to the different monitoring indicators, this study was divided into two programs. (1) The rats were studied at 28 days postoperatively and nerve function, survival rate and weight change percentages were measured; (2) rats were sacrificed 48 h after injury and samples were harvested for subsequent measurements, including measurements of the infarct volume, brain water content, inflammatory cells and factors, catecholamine levels, acetylcholine levels, immunohistochemical staining, and western blotting.

### Diabetic Rat Model

We fed rats a high-fat diet consisting of 20% lard, 20% sucrose, 2.5% cholesterol and 57.5% standard diet for 4 weeks to induce diabetes (DM) (Reed et al. 2000; Duca et al. 2015). Then, the rats were allowed to drink freely overnight and STZ (streptozotocin) dissolved in citric acid-citrate buffer (pH 4.5; intraperitoneal injection, 35 mg/kg; Sigma–Aldrich (Shanghai) Trading Co., Ltd., Shanghai, China) Zotobacter) was injected. Seven days later, blood glucose levels were randomly detected. Animals with a level ≥ 11.1 mmol/l were selected as diabetic rats. The blood glucose level of diabetic rats in this study was 15–25 mmol/l.

### MCAO Rat Model

We established a rat model of middle cerebral artery occlusion (MCAO) (Belayev et al. 1996). Experimental rats were administered 5% sevoflurane (Baxter, USA) through a face mask for the induction of anaesthesia, 2–3% sevoflurane was used for anaesthesia maintenance, and spontaneous breathing was retained. After preparation and disinfection of the skin in the operation area and local infiltration anaesthesia with 1% lidocaine, a midpoint incision of approximately 0.5 cm was made at the midpoint of the craniopia of both ears to identify the fontanelle, where the anterior fontanelle was defined as zero. The cerebral blood flow probe was fixed on the left side of the fontanelle at 4 mm and 1.5 mm, and the cerebral blood flow (CBF) was monitored with a laser Doppler blood flow metre (TF5000; PRIMED AB; Stockholm; Sweden). The animal was slowly turned over, and the neck skin was prepared and disinfected. Local infiltration anaesthesia was induced with 1% lidocaine. An incision was created 3 mm parallel to the midline, micro tweezers were used to bluntly separate the subcutaneous tissue, the left sternocleidomastoid muscle was exposed, and the first sternocleidomastoid muscle was gradually freed. The left carotid sheath can be seen between the second abdominal muscles, sternocleidomastoid muscle and scapula-thyroid muscle. The common carotid artery, external carotid artery and internal carotid artery were bluntly separated. Electrocoagulation disconnected the communication branch between the external carotid artery and the internal carotid artery and the accessory branch of the external carotid artery, the left external carotid artery was ligated and disconnected, the proximal end of the common carotid artery and the distal heart of the internal carotid artery was clamped with an arterial clip, and a suture (Doccol #4035, #4037, and #4039, USA) was inserted from the stump of the external carotid artery. The cerebral blood flow suddenly decreased to a value greater than 70% of the basal level. The incision was sutured. The plug insertion time and cerebral blood flow value were recorded. For rats with a Longa score of 2 (Belayev et al. 1996), the thread plug was removed after 90 min of embolization. After the operation, the animals were sent to the PACU to rouse from anaesthesia and remain warm.

### Molecular Hydrogen Preparation

Hydrogen/oxygen mixture therapy was delivered using a novel device, Hydrogen/Oxygen Generator with Nebulizer (AMS-H-01, Shanghai Asclepius Meditech Co., Ltd., China), at a flow rate of 3.0 L/min and hydrogen/oxygen volume ratio of 2:1. In addition, the gas ratio was 42% H_2_-21% O_2_-37% N_2_. The mixed gas was output to an explosion-proof box. The explosion-proof box contained a gas absorption part, an inhalation and exhalation gas circuit, and an exhaust gas absorption device. It was also equipped with a safety alarm and a hydrogen concentration detection metre.

### Molecular Hydrogen Therapy

Rats inhaled molecular hydrogen for 60 min beginning 20 min after MCAO modelling and daily after MCAO/R. The treatment time of the H_2_ daytime treatment group was 9:00–11:30, and the treatment time of the H_2_ night-time treatment group was 16:30–19:00.

### Measurement of Blood Sugar Levels

Thirty minutes before the establishment of the MCAO model in diabetic rats and 15 min after MCAO/R, blood was collected from the tail vein of the rats, and the blood glucose level was measured and recorded.

### Assessment of Neurological Outcomes, Survival Rate, and Weight Change Percentage

Neurological function was measured in rats on the 1st, 3rd, 5th, 7th, 14th, and 28th days after surgery with the modified nervous system severity score (mNSS) (Chen et al. 2001) and the Garcia test, and the 28-day survival rate was recorded. All rats received two adaptation training sessions before surgery. The evaluation was conducted by the same researcher and supervised by the same person under mutual blinding conditions. Body weight was measured daily for 28 days, and the percentage of weight change relative to the baseline body weight was determined (n = 6 rats/group).

### Infarct Volume Measurement

After 48 h of reperfusion, the rats were sacrificed with a lethal dose of sodium pentobarbital. The rat brain was quickly removed, cut into 2 mm coronal sections, stained with 1% 2,3,5-triphenyltetrazolium chloride (TTC, Biotosharp, Beijing, China) at 37 °C for 20 min, and then incubated at 4 °C overnight with 1% paraformaldehyde (Chi et al. 2018; Li et al. 2019). ImageJ software was used to measure the infarct and hemisphere areas. The ratio of hemisphere infarction was calculated as (area of the contralateral hemisphere -area of the ipsilateral hemisphere without infarction)/area of the contralateral hemisphere (n = 6 rats/group).

### Measurement of the Brain Water Content

After 48 h of reperfusion, we randomly selected 6 rats from each group and estimated the brain water content (Li et al. 2020). Briefly, we separated the cerebral hemisphere on the ischaemic side from other parts and recorded the weight (wet weight). Next, each specimen was dried in an electric furnace at 80 °C for 48 h, and the weight (dry weight) was recorded. We used the following formula to calculate brain water content: brain water content = (wet weight-dry weight)/wet weight × 100%.

### Measurement of Inflammatory Cytokine, Catecholamines and Acetylcholine Levels

The rats were anaesthetized with pentobarbital, and the femoral artery was punctured to collect blood. The blood sample was placed in a heparin-coated tube and centrifuged (3500 rpm, 4 °C, 20 min). The plasma levels of IL-6, IL-1β, TNF-α, epinephrine, norepinephrine, dopamine and acetylcholine were measured 48 h after reperfusion. We used enzyme-linked immunosorbent assay (ELISA) kits to analyse the levels of each protein (#rat IL-6, #rat IL-1β and #rat TNF-α ELISA kit, Boster, China; # Rat Adrenaline, # Rat Norepinephrine, # Rat Dopamine and # Rat Acetylcholine, Langton, China) according to the manufacturer’s instructions (n = 6 rats/group).

### Immunofluorescence Measurement

After 48 h of reperfusion, the rats were euthanized with a lethal dose of sodium pentobarbital and perfused transcardiacally with normal saline and 4% paraformaldehyde. The brain was postfixed with 4% paraformaldehyde at 4 °C for 24 h. The infarct region was cut into 10 mm transverse frozen sections. Sections were washed with 0.1% Triton X-100 for 20 min and then blocked with 3% BSA for 30 min. The sections were incubated with primary antibodies against TLR4 (1:100; BS3489, Bioworld Technologies) and NF-κB (1:200; BMS-33117 M, Bioss) at 4 °C overnight and then incubated with FITC-conjugated secondary antibodies for 50 min. Finally, the sections were incubated with DAPI for 10 min and then visualized with an optical microscope (Nikon Eclipse Ti-SR, Japan) at 400 × magnification. Merged images were captured under the same contrast settings: TLR4 is shown in green, NF-κB is shown in red, and the nucleus is shown in blue (n = 6 rats/group).

### Western Blot Assay

After 48 h of reperfusion, the cerebral cortex was homogenized and centrifuged at 14,000 rpm for 20 min at 4 °C. We collected the supernatant and tested the protein concentration with the BCA test kit. Then, we separated the protein samples on 10% SDS–PAGE gels and transferred them to a nitrocellulose membrane. The membrane was blocked with 5% milk powder in 0.1% TBS-T, and primary antibodies against NF-κB p65 (1:1000; #48,498, SAB), β-Actin (1:400; PR-0255, ZSGB Bio), and phospho-NF-κB p65 (phospho Ser536; 1:2000; YP0191, Immunoway) were incubated with the membrane at 4 °C overnight. Subsequently, we washed the membrane with TBS-T and incubated it with a goat anti-rabbit horseradish peroxidase-conjugated secondary antibody (1:5000; ZB-2301, ZSGB Bio) for one hour. An electrochemiluminescence kit was used to detect the protein bands, and a gel imaging system was used for data collection and analysis (n = 6 rats/group).

### Statistical Analysis

The sample size was based on our previously reported research. No data were missing, and no data were excluded from the analysis. We used SPSS (version 21.0) statistical software and GraphPad Prism 9 software to analyse the data. Data are presented as the means ± standard errors of the means (SEM). The normality of the distribution was assessed using the Shapiro–Wilk normality test, and a smaller sample size distribution was considered normal. Two groups were compared using t tests or Mann–Whitney U tests. One-way analysis of variance (ANOVA) was used to compare multiple groups, and then the Student-Newman–Keuls test was used. Nonparametric tests were used to compare the differences between the tested groups. Kaplan–Meier survival curves were compared using the log-rank test. Repeated measures analysis of variance was used for comparisons within the same group. A p value < 0.05 was considered statistically significant.

## Results

### Blood Sugar Levels

During the operation of diabetic rats, the blood sugar level increased significantly. Rapid changes in blood glucose levels in a short time are the main cause of blood glucose fluctuations in diabetic rats (Table 1).

**Table 1:** Changes in blood glucose of MCAO and H_2_ treatment group

Group	M	H
preoperative	18.9 ± 4.03*	18.4 ± 3.32
postoperative	23.75 ± 4.25	15.9 ± 4.03

*p < 0.05 vs group MCAO postoperative

### Molecular Hydrogen Reduces the Infarct Volume and Brain Water Content

**Fig. 2:**
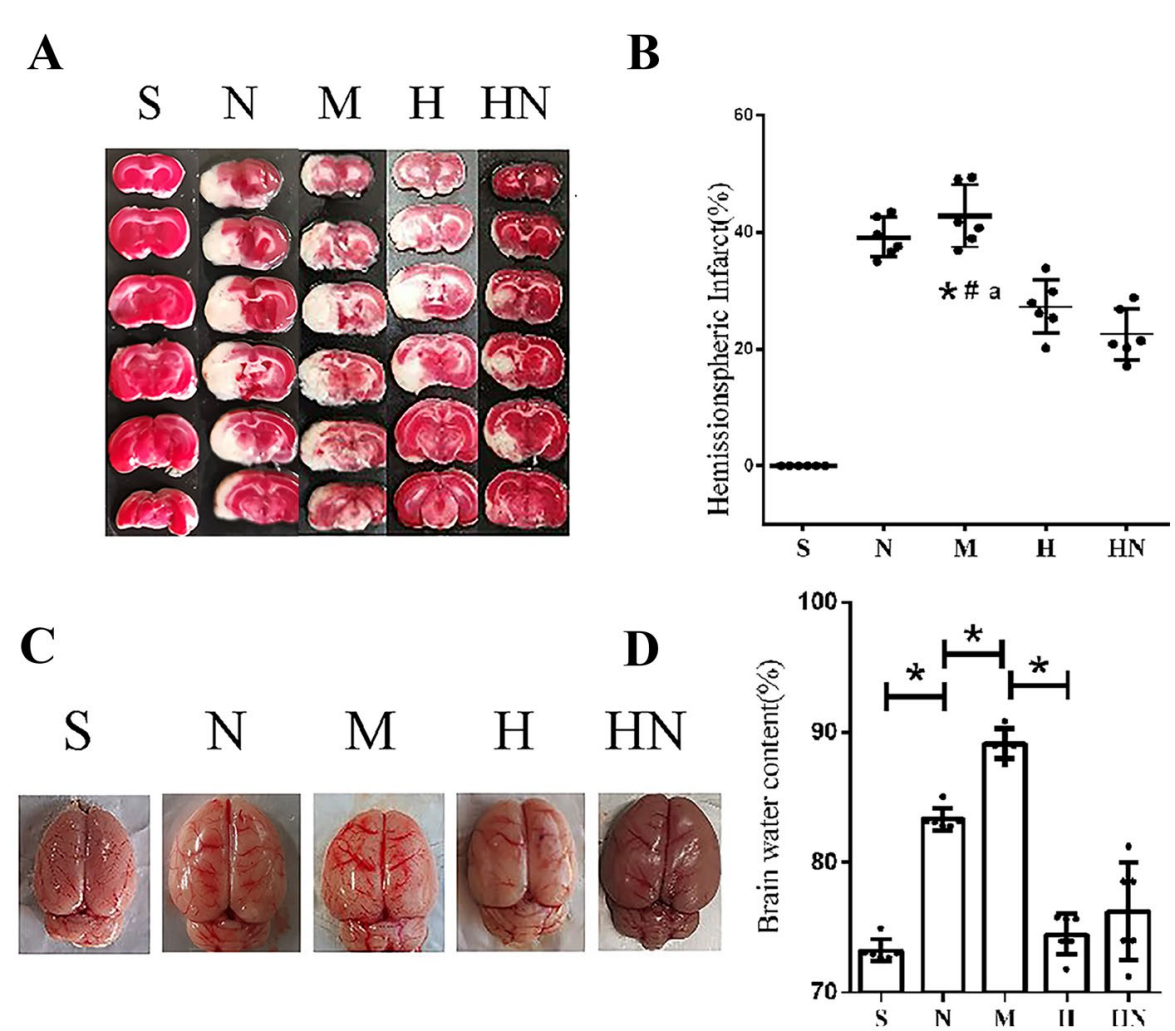
Infarct volume and brain oedema. **A** TTC staining. **B** Statistical analysis of TTC staining in each group (n = 6). *p < 0.05 compared with the Sham group; #p < 0.05 compared with the H_2_ daytime treatment group; ap < 0.05 compared with the H_2_ night-time treatment group. **C** Gross map of the brain tissue. **D** Statistical analysis of the brain water content in each group (n = 6). *p < 0.05

As shown in Fig. 2A, B, the infarct area of rats in group H and group HN was significantly improved at 48 h after MCAO. Diabetes significant exacerbated brain water content after stroke and significantly improved in group H and group HN (Fig. 2C, D). The effect of hydrogen on ameliorating ischaemic stroke was not influenced by the circadian rhythm.

### The Effect of Molecular Hydrogen on Nervous System Outcomes, Weight Change Percentage and Survival Rate

**Fig. 3:**
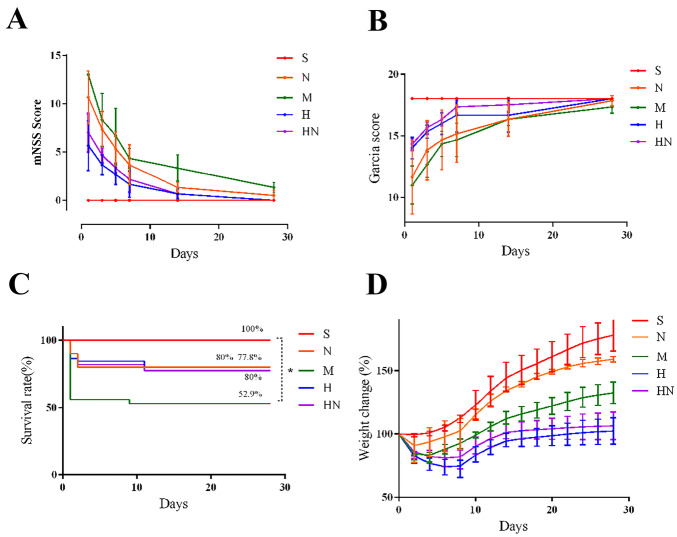
Neurological outcomes, survival rates, and weight change percentages. **A** Scores of neurological function (mNSS). **B** Scores of neurological function (Garcia). **C** Survival of rats in each group. **D** Weight change rate of rats in each group (n = 6). *p < 0.05

Diabetes significant exacerbated post-stroke neurological deficits and poor prognosis (Fig. 3A−D). After molecular hydrogen treatment, the neurological deficits of diabetic stroke rats were significantly reduced, and no significant difference was observed between group H and group HN (Fig. 3A, B). Compared with rats in group M (52.9%), molecular hydrogen treatment induced significant survival benefits, group H (80%) and group HN (77.8%) survived to the 28th day (P < 0.05). No significant difference was observed between group H and group HN (Fig. 3C). In terms of postoperative weight changes, the weight of group M reached the lowest value on the 5th day and returned to the baseline level on the 11th day. The weight of group H and group HN reached the lowest value on the 8th day and returned to the baseline level on the 17th day (Fig. 3D).

### The Effect of Molecular Hydrogen on Epinephrine, Norepinephrine, Dopamine and Acetylcholine Levels

**Fig. 4:**
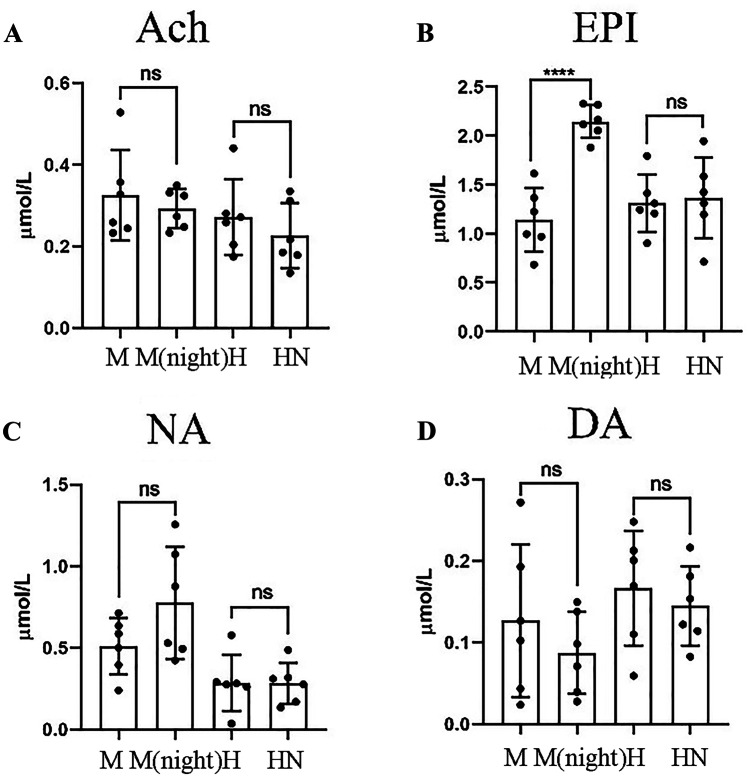
Catecholamine and acetylcholine levels. **A** The difference in Ach levels between the day and night in rats from each group. **B** The difference in EPI levels between the day and night in rats from each group. **C** The difference in NA levels between the day and night in rats from each group. **D** The difference in DA levels between the day and night in rats from each group (n = 6). Two-tailed t tests were used for comparisons between groups. *p < 0.05

After molecular hydrogen treatment, the autonomic nerves tend to be balanced. Significant circadian rhythm-related differences in acetylcholine levels were observed in rats. After molecular hydrogen treatment, this difference was significantly reduced and balanced (Fig. 4A−D). Thus, the protective effect of hydrogen on MCAO-induced damage independent of circadian rhythms may be related to the regulation of autonomic homeostasis.

### The Effect of Molecular Hydrogen on Brain Oedema and Systemic Inflammation

**Fig. 5:**
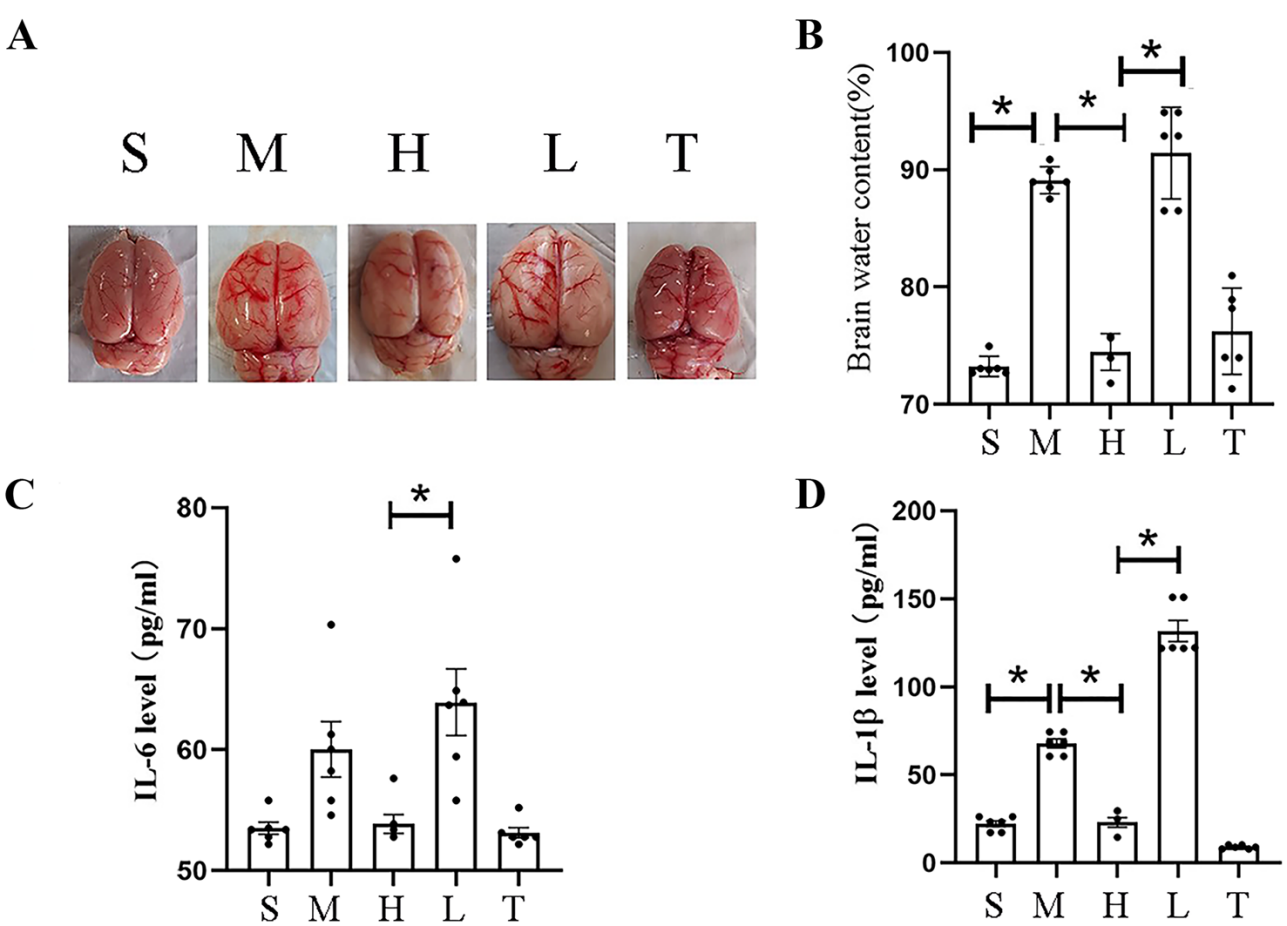
Levels of inflammatory factors and brain water content. **A** Gross map of the brain tissue. **B** Statistical analysis of the brain water content in each group. **C** IL-6 levels in each group. **D** IL-1β levels in each group (n = 6). Two-tailed t tests were used for comparisons between groups. *p < 0.05

The enhanced effects of inflammation were reduced by molecular hydrogen treatment at attenuating acute cerebral oedema (Fig. 5A, B) and significantly reduced the level of the inflammatory cytokine IL-1β (Fig. 5C, D). Hydrogen treatment after TLR4 pathway agonism did not show a therapeutic advantage with an anti-inflammatory effect. The protective effect may be closely related to the TLR4/NF-κB pathway.

### The Effect of Molecular Hydrogen on the TLR4/NF-κB Pathway

**Fig. 6:**
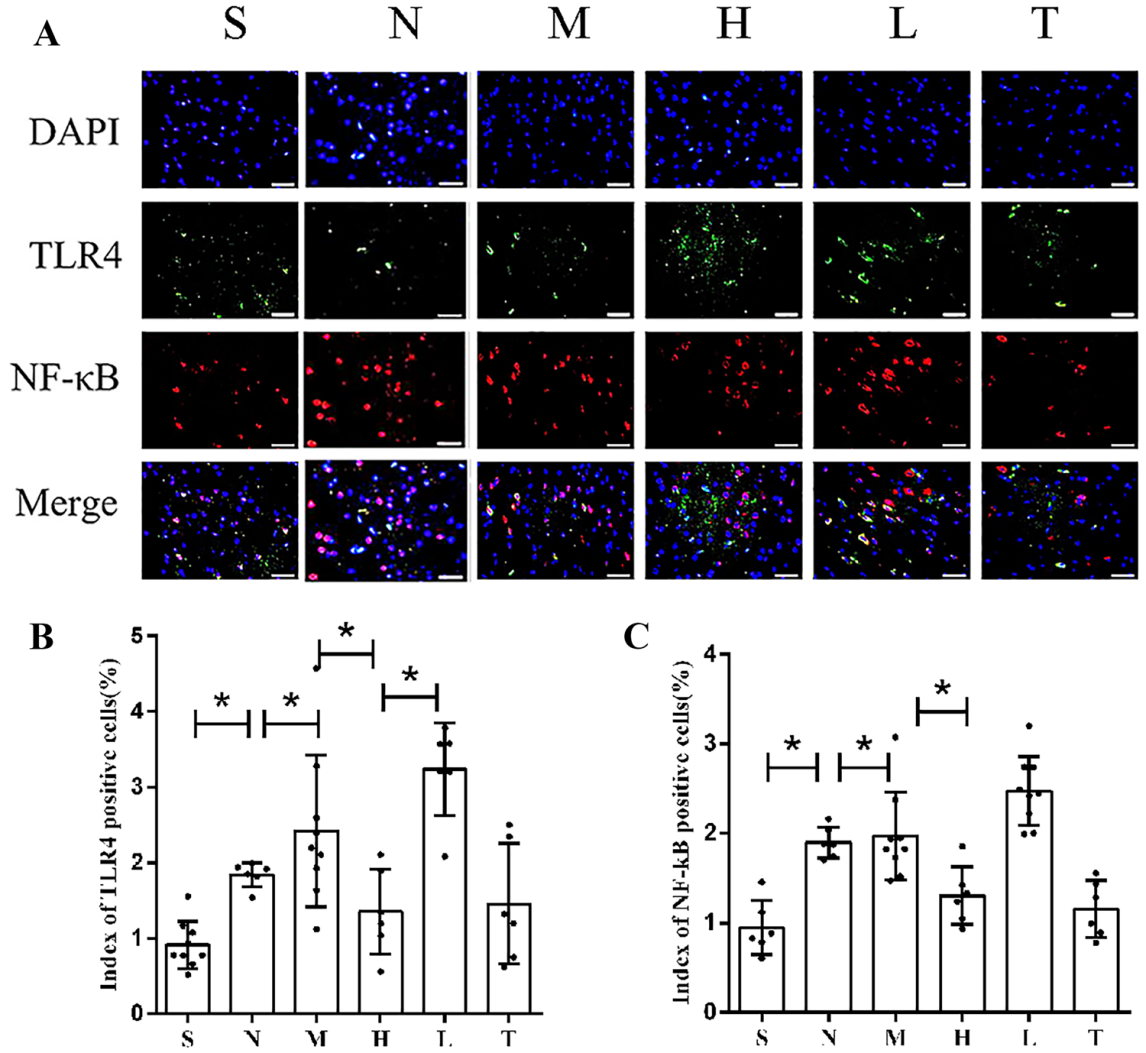
Immunofluorescence staining. **A** Images of immunofluorescence staining. **B** Statistical analysis of TLR4 levels in the ischaemic area of rats in each group. **C** Statistical analysis of NF-κB levels in the ischaemic area of rats in each group (n = 6). Two-tailed t tests were used for comparisons between groups. *p < 0.05

**Fig. 7:**
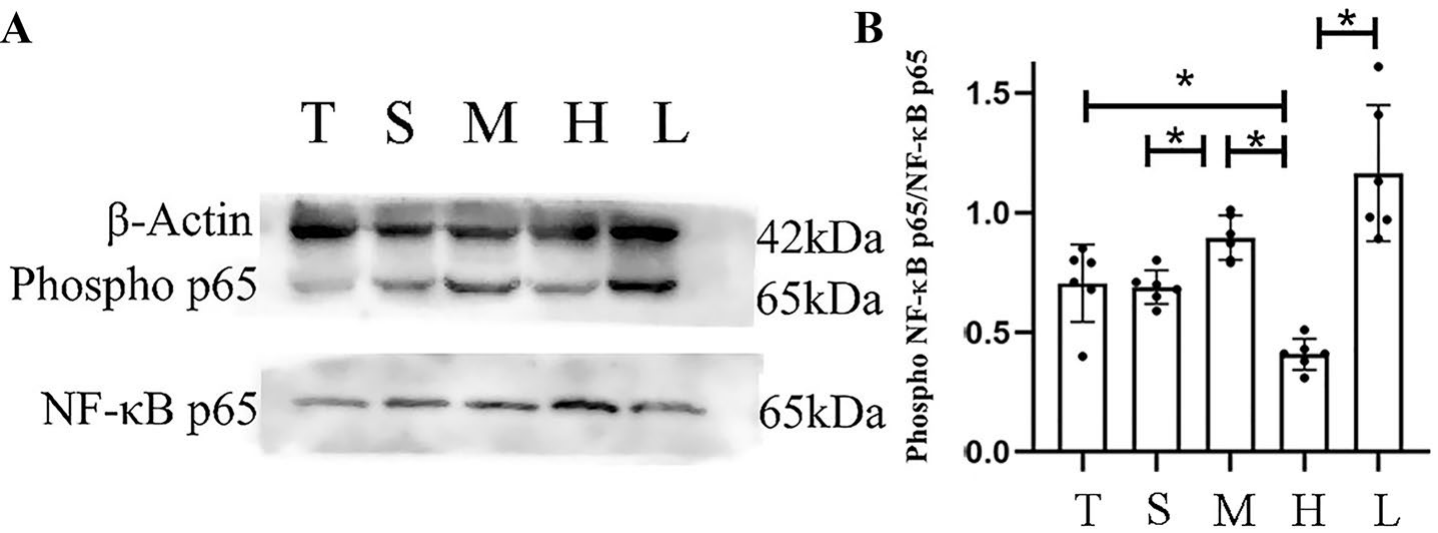
Western blot. **A** Western-blots showing p-NF-κB and NF-κB levels in each group. **B** Statistical analysis of the relative level of p-NF-κB among total NF-κB in the ischaemic area of rats in each group (n = 6). Two-tailed t tests were used for comparisons between groups. *p < 0.05

The western blot method was used to detect the levels of NF-κB p65 and phosphorylated p65. Diabetes caused significant activation of the TLR4/NF-κB pathway after stroke (Fig. 6A−C). After molecular hydrogen treatment, the phosphorylation of NF-κB p65 was inhibited, and the systemic inflammatory response was reduced in diabetic stroke rats (Fig. 7A, B). While analysing the immunofluorescence staining, we also quantitatively observed much higher TLR4 expression in group M than that in group S, which was significantly reduced after molecular hydrogen treatment, and this change disappeared after the administration of LPS. Immunofluorescence staining showed that NF-κB was mainly located in the nucleus, consistent with the results observed for TLR4 (Fig. 6A−C). The mechanism underlying the neuroprotective effect of molecular hydrogen was associated with inhibition of TLR4/NF-κB pathway activation.

## Discussion

The care of patients with diabetes who experience an ischaemic stroke presents an important challenge in neurointensive care units. A key issue to be solved in the clinic is to seek effective and improved methods to ensure patient survival and quality. Undoubtedly, molecular hydrogen improves the ischaemic penumbra, but the effect and mechanism of hydrogen on stroke in patients with diabetes are not clear (Chen et al. 2019; Li et al. 2019; Jiang et al. 2020). As shown in our previous study, diabetes aggravates acute cerebral oedema and systemic inflammation, which may be the key to the short survival and poor long-term neurological outcomes after stroke in patients with diabetes (Li et al. 2020). In the present study, molecular hydrogen effectively alleviated the abovementioned pathological changes, and no diurnal difference in this improvement was observed.

The circadian rhythm is an important cause of the failure of the basic research results for ischaemic stroke to be translated into clinical practice. Studies have shown that many treatments are effective in rodents during the inactive period during the day, but they show no improvement in the active period at night. As shown in the present study, circadian rhythm did not affect the treatment effect of molecular hydrogen on stroke in diabetic rats. We speculate that this difference may be related to adjusting the biological clock or interfering with the balance of autonomic nerves. By detecting catecholamine and norepinephrine levels during the day and at night, we speculate that the dynamic balance of sympathetic, parasympathetic, and vague nerves is always maintained in animals treated with molecular hydrogen, which suppresses the nocturnal sympathetic active phase of rats and the risk of damaging blood vessels to ensure the effectiveness of treatment. This lack of difference between different periods of the circadian rhythm also provides a basis for the clinical transformation of molecular hydrogen.

Diabetes is an independent risk factor for stroke. The pathological exacerbation of hyperglycaemia in the acute phase of stroke and its accompanying series of pathological changes are unique risk conditions for diabetic stroke patients (Johnston et al. 2019; Lo et al. 2020; Cukierman-Yaffe et al. 2021). We summarized the results of previous studies and found that neurological dysfunction and mortality increased significantly after stroke in patients with diabetes, accompanied by cerebral oedema and systemic inflammation, which may be a key factor contributing to death in the acute phase. Systemic inflammation may cause or aggravate local or overall brain inflammation, while global brain inflammation is closely related to the aggravation of long-term neurological dysfunction (Shi et al. 2019). In the present study, molecular hydrogen effectively improved the long-term neurological function and survival of rats and reduced cerebral oedema. In addition, serum levels of IL-6, IL-1β and TNF-α, indicators of systemic inflammation (Ogawa 2017; Rana et al. 2018; Zhang et al. 2018; Huggard et al. 2020), were also reduced after the administration of molecular hydrogen. The TLR4/NF-κB p65 pathway, an upstream pathway that regulates IL-6, IL-1β and TNF-α levels, reflects inflammatory changes at the molecular level (Shang et al. 2019; Fu et al. 2021). Through the detection of changes in blood glucose levels, brain oedema, and levels of inflammatory factors and other indicators, we propose that the mechanism by which molecular hydrogen improves diabetic stroke may be related to improving oedema, pathological hyperglycaemia and systemic inflammation after TLR4/NF-κB pathway activation. By improving these conditions and alleviating the pressure of acute disease, molecular hydrogen may also be the key to clinical intervention for patients with diabetes who experience a severe stroke to obtain prolonged survival and a better prognosis.

In addition, in this study, rats in the molecular hydrogen treatment group gained weight more slowly, and their recovery trend did not meet the expectations. Diabetic rats present typical signs of obesity, and the rats eat and drink freely. Previous studies have shown that molecular hydrogen exerts a certain weight loss effect, and its mechanism is related to increasing metabolism, lowering blood sugar levels, and improving gluconeogenesis (Kamimura et al. 2011; Botek et al. 2019; LeBaron et al. 2020). In the present study, the blood sugar level of rats was reduced after inhaling molecular hydrogen, and this hypoglycaemic effect may be related to the slow weight gain. On the other hand, although diabetic stroke is a major component of critical neurological illness, the mortality rate of rats in this study is still relatively high compared with the actual value of clinical patients. The possible reasons are described below. First, clinical patients with diabetes are treated with hypoglycaemic agents, but the rats were not treated with insulin. Second, clinical patients can be provided parenteral nutrition after surgery to facilitate early recovery, while the diet of rats was not changed, and no additional nutritional support was provided after surgery. Finally, clinical patients received a series of symptomatic and supportive measures in real time, including anti-inflammatory agents, whereas rats were not administered additional antibiotics. The long-term indicators of rats show the advantages of molecular hydrogen for improving long-term neurological outcomes, and the increase in the survival rate reflects the prospects for the clinical application of molecular hydrogen. However, there was also death in group H rats. Although this study found that molecular hydrogen effectively improved cerebral ischemia and edema, there is no evidence shows H_2_ could reverse the damage such as haemorrhage caused by reperfusion. We speculate that this may be the cause of H group rats’ death.

In summary, this study explores the role of molecular hydrogen from the perspective of circadian rhythms and provides a transformational basis for its clinical application for diabetic stroke. Exploring the mechanism from the perspective of molecular pathways provides a theoretical basis for related basic research. However, at the same time, the immunological mechanisms of stroke and the changes in immune balance in the pathological state of diabetes are more complicated, and we have not explored these processes in depth; treatment with molecular hydrogen is also lacking big data for clinical applications. Therefore, further research on molecular hydrogen is still needed to clarify clinical and basic aspects.

## Supplementary Information

Below is the link to the electronic supplementary material.

**Fig. 1** Original image of the Western blot. (PDF 40 kb)

**Fig. 2** Ethical review. (PDF 228 kb)
