# Supplementary figures and images for: Molecular Hydrogen Mediates Neurorestorative Effects After Stroke in Diabetic Rats: the TLR4/NF-κB Inflammatory Pathway
Source: J Neuroimmune Pharmacol. 2022 Jul 27;18(1-2):90–9. doi: 10.1007/s11481-022-10051-w (PMC10485112; doi:10.1007/s11481-022-10051-w)

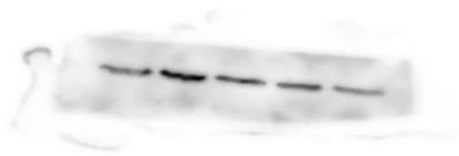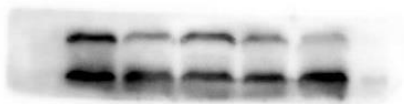

**Attached Fig.1** Western-blot gel original image.

Supplement: Supplementary file 1 — Fig. 1 Original image of the Western blot. (PDF 40 kb) [file 11481_2022_10051_MOESM1_ESM.pdf]
