# Supplementary material for: Molecular Hydrogen Mediates Neurorestorative Effects After Stroke in Diabetic Rats: the TLR4/NF-κB Inflammatory Pathway
Source: J Neuroimmune Pharmacol. 2022 Jul 27;18(1-2):90–9. doi: 10.1007/s11481-022-10051-w (PMC10485112; doi:10.1007/s11481-022-10051-w)
Supplement: Supplementary file 2 — Fig. 2 Ethical review. (PDF 228 kb) [file 11481_2022_10051_MOESM2_ESM.pdf]

Appendices 4.

Ethical review

哈尔滨医科大学附属第二医院医学伦理委员会

动物实验伦理审查批件

伦理审查批件号：SYDW2020-032

项目名称	吸入高浓度氢气对糖尿病大鼠脑缺血再灌注损伤的影响及机制研究		
科室	麻醉科	项目来源	自选
项目负责人	杨万超	职 称	主任医师
研究起止时间	2020 年 10 月 14 日—— 2021 年 2 月 30 日		
审查方式	□会议审查 √快速审查		
审查文件	1、动物实验伦理审查申请表<br>2、研究方案（版本号：1.0 日期：2020 年 10 月 16 日 ）<br>3、课题申报书		
审查结论			
根据《实验动物管理条例》、《关于善待实验动物的指导性意见》、《黑龙江省实验动物管理条例》等相关规定，哈尔滨医科大学附属第二医院医学伦理委员会对该项目进行了实验动物保护和福利伦理审查后认为：该项目在方案设计中符合实验动物保护和福利伦理原则，同意在课题立项后，项目负责人遵照提交给伦理委员会的研究方案开展研究工作。			
审查意见	同意		
正（副）主任委员签字	[illegible]	日期	2020 年 11 月 10 日
伦理委员会	哈尔滨医科大学附属第二医院医学伦理委员会（盖章）		
